# The association between tyrosine kinase inhibitors and fatal arrhythmia in patients with non-small cell lung cancer in Taiwan

**DOI:** 10.3389/fonc.2023.1172036

**Published:** 2023-04-17

**Authors:** Wei-Ting Chang, Hui-Wen Lin, Ting-Chia Chang, Sheng-Hsiang Lin, Yi-Heng Li

**Affiliations:** ^1^Institute of Clinical Medicine, College of Medicine, National Cheng Kung University, Tainan, Taiwan; ^2^Division of Cardiology, Department of Internal Medicine, Chi-Mei Medical Center, Tainan, Taiwan; ^3^Department of Biotechnology, Southern Taiwan University of Science and Technology, Tainan, Taiwan; ^4^Department of Internal Medicine, National Cheng Kung University Hospital, College of Medicine, National Cheng Kung University, Tainan, Taiwan; ^5^Biostatistics Consulting Center, National Cheng Kung University Hospital, College of Medicine, National Cheng Kung University, Tainan, Taiwan; ^6^Division of Pulmonology, Department of Internal Medicine, Chi Mei Medical Center, Tainan, Taiwan; ^7^Department of Public Health, College of Medicine, National Cheng Kung University, Tainan, Taiwan

**Keywords:** NSCLC, tyrosine kinase inhibitors, platinum analogues, death, arrhythmia

## Abstract

**Objective:**

As a standard therapy, tyrosine kinase inhibitors (TKIs) improved survival in patients with non-small cell lung cancer (NSCLC) and epidermal growth factor receptor (EGFR) mutation. However, treatment-related cardiotoxicity, particularly arrhythmia, cannot be ignored. With the prevalence of EGFR mutations in Asian populations, the risk of arrhythmia among patients with NSCLC remains unclear.

**Methods:**

Using data from the Taiwanese National Health Insurance Research Database and National Cancer Registry, we identified patients with NSCLC from 2001 to 2014. Using Cox proportional hazards models, we analyzed outcomes of death and arrhythmia, including ventricular arrhythmia (VA), sudden cardiac death (SCD), and atrial fibrillation (AF). The follow-up duration was three years.

**Results:**

In total, 3876 patients with NSCLC treated with TKIs were matched to 3876 patients treated with platinum analogues. After adjusting for age, sex, comorbidities, and anticancer and cardiovascular therapies, patients receiving TKIs had a significantly lower risk of death (adjusted HR: 0.767; CI: 0.729–0.807, p < 0.001) than those receiving platinum analogues. Given that approximately 80% of the studied population reached the endpoint of mortality, we also adjusted for mortality as a competing risk. Notably, we observed significantly increased risks of both VA (adjusted sHR: 2.328; CI: 1.592–3.404, p < 0.001) and SCD (adjusted sHR: 1.316; CI: 1.041–1.663, p = 0.022) among TKI users compared with platinum analogue users. Conversely, the risk of AF was similar between the two groups. In the subgroup analysis, the increasing risk of VA/SCD persisted regardless of sex and most cardiovascular comorbidities.

**Conclusions:**

Collectively, we highlighted a higher risk of VA/SCD in TKI users than in patients receiving platinum analogues. Further research is needed to validate these findings.

## Introduction

With improvements in anticancer therapies, the overall survival of patients with advanced non-small cell lung cancer (NSCLC) has improved (1, 2). In the early years, platinum-based chemotherapy was the standard treatment for patients with advanced NSCLC (3, 4). However, it improves the five-year survival rate to only 3.5% (1, 5). In addition, platinum analogues, including cisplatin and carboplatin, have been observed to have potential nephrotoxicity, neurotoxicity, and cardiotoxicity (3, 4). In contrast, tyrosine kinase inhibitors (TKIs) have dramatically increased the survival of patients with epidermal growth factor receptor (EGFR) mutation-positive NSCLC (6, 7). Lin et al. showed that the five-year survival rate of patients with EGFR-mutant metastatic NSCLC increased to 14.6% (2). Notably, EGFR mutations account for 10% of patients in the United States, 12.8% in Europe, and up to 50% in Asia (8). This has brought awareness to a potential issue with the widespread prescription of TKIs in Asia. Nevertheless, TKIs may exacerbate myocardial ischemia, heart failure, hypertension, and QT prolongation (9, 10). Among these complications, QT prolongation-induced fatal arrhythmia requires the most attention (9–11). However, there is a lack of comparison between platinum analogues and TKIs regarding their long-term effects on arrhythmia. Therefore, we assessed the risks of death and arrhythmia, including ventricular arrhythmia (VA), sudden cardiac death (SCD), and atrial fibrillation (AF), among patients with NSCLCs who use platinum analogues compared with those who use TKIs in a national cohort.

## Methods

### Patients and study design

Using the Taiwanese National Health Insurance Research Database (NHIRD) and National Cancer Registry, we observed patients with NSCLC from 2001 to 2014. Given that the prescription of TKIs depends on whether the patients with NSCLC had EGFR mutations, to exclude selection bias between TKI users and non-users, which may skew the results, we randomly selected a control group during 2001–2004, the period when TKIs were not yet available to match with the study cohort of TKI use. TKIs included erlotinib, afatinib and gefitinib during 2005–2014. The control group consisted of patients who had received platinum analogues treatment, including cisplatin and carboplatin, and were propensity score-matched 1:1 to patients who had received TKI treatment for more than 90% of the time in the subsequent 30 days following enrollment. Patients with a history of NSCLC, aged < 18 years, without complete data, who used platinum analogues or TKIs within a year before the index date, or with medical records after death were excluded. The index date was set as the first day of TKI or platinum analogue use. The data used in this study were obtained from the original claims database for the reimbursement of all Taiwanese residents from the NHIRD (12, 13). The accuracy of NHIRD has been validated in previous studies (12, 13). The flowchart of the study design is shown in **Supplemental Figure 1**. From the database, details on the patient’s age, sex, medical history, concurrent drugs taken within the past three months, and treatments or procedures were taken. Before 2015, the diagnostic codes in the NHIRD were identified using the International Classification of Diseases, Ninth Revision, Clinical Modification (ICD-9-CM), while after 2016, they were changed to the International Classification of Diseases, Tenth Revision, Clinical Modification (ICD-10-CM). Within the NHIRD, continuous claims data can be tracked for the same patient. The International Classification of Disease (ICD) diagnosis and treatment codes are presented in **Supplemental Table 1**. This study was approved by our institutional review board (IRB A-EX-111-003; CV code: 10406-E01), and the requirement for informed consent was waived owing to the retrospective study design.

### Study endpoint

The primary endpoint was death, while arrhythmias, VA, including ventricular tachycardia and fibrillation, SCD, and AF, were the main consequences. All patients were followed from the index date to death or were lost to follow-up. As ICD-9-CM was replaced by ICD-10-CM by the Taiwan National Health Insurance in 2016, both ICD-9 and 10 codes were employed to identify endpoints in the primary outcome during the follow-up. The follow-up period was three years.

### Statistical analysis

Continuous variables are represented as mean ± standard deviation, and categorical variables are presented as numbers and percentages. Propensity score analysis was performed to reduce any selection bias caused by variations in the clinical characteristics between groups despite the nonrandomized nature of the trial (14). The propensity score is defined as the probability of exposure to treatment, conditional on the baseline characteristics of the study subjects. In this case, the propensity score for receiving platinum analogues or TKIs was calculated using multivariate logistic regression analysis, subject to the factors index year, age, sex, procedures, drugs, and comorbidities before enrollment. Instead of statistical testing, absolute standardized mean difference (ASMD) was used to compare the distributions of clinical features between the two groups. An ASMD of 0.1 denotes an insignificant difference between the two groups. The ASMD was calculated as the mean or proportion of a variable divided by the pooled estimate of the standard deviation of that variable. Using a multivariate Cox proportional hazards model, we analyzed the relationship between the endpoints and different treatments. All potential confounders were considered when calculating the HRs and their 95% CIs from the Cox models. Considering that death may reduce the incidence of cardiovascular events, the competing risk method (subdistribution HR [sHR]) was also used to calculate the risk of arrhythmia from the Cox regression model after adjusting for all potential confounders. Differences between the groups were compared using Gray’s test. The findings were plotted using the cumulative incidence function for outcome events and competing risk events of death. To estimate P-values for interactions in the subgroup analysis, we utilized the same Cox proportional hazards model (competing risk technique). All analyses of the data were performed using SAS 9.4 for Windows (SAS Institute Inc., Cary, NC).

## Results

### Demographic characteristics of patients with NSCLC

We initially identified 10582 patients with NSCLC receiving TKIs and 7883 patients treated with platinum analogues. After adjusting for age, sex, comorbidities, and anticancer and cardiovascular therapies, 3876 TKI users were 1:1 matched with 3876 patients treated with platinum analogues. We observed that the ages (average age, 66 years) and sexes of the two groups were similar (**Table 1**). Only a small portion of patients received radiotherapy or surgery for anticancer therapies. Among the studied patients, approximately 40% had a history of hypertension or chronic obstructive lung disease/asthma, while approximately 15% had diabetes or hyperlipidemia. One-quarter of the studied patients were prescribed ACEIs/ARBs, whereas approximately one-fifth of them received beta-blockers or antiplatelet agents. As illustrated in **Supplemental Table 2**, in addition to TKIs and platinum analogues, approximately 10% of patients were also treated with taxanes. Gemcitabine, an inhibitor of DNA synthesis, was prescribed to 42% of platinum analogue users compared with 11% of TKI users. Fifteen percent of platinum analogue users, compared with 34% of TKI users, subsequently received vinorelbine, an antimitotic anticancer agent. The details of anti-arrhythmic drugs are listed in **Supplemental Table 3**, and only a small number of patients were on these drugs.

**Table 1:** The baseline characteristics of patients with non-small cell lung cancer (NSCLC) treated with either Platinum analogues or TKIs before and after propensity score matching.

	Before propensity score matching							After propensity score matching						
	Total N=18465		Platinum analogues N=7883		TKIs N=10582		ASMD	Total N=7752		Platinum analogues N=3876		TKIs N=3876		ASMD
Age							0.549							0.112
Mean (SD)	66.38	(12.34)	62.65	(11.46)	69.16	(12.23)		66.05	(12.14)	65.37	(10.64)	66.73	(13.44)	
Median (IQR)	68.00	(18.00)	64.00	(17.00)	71.00	(19.00)		68.00	(17.00)	67.00	(14.00)	70.00	(21.00)	
Sex														0.067
Male, N (%)	9359	(50.69)	5171	(65.60)	4188	(39.58)	0.540	4511	(58.19)	2192	(56.55)	2319	(59.83)	
Anti-cancer therapies														
Radiotherapy, N (%)	497	(2.69)	214	(2.71)	283	(2.67)	0.003	253	(3.26)	125	(3.22)	128	(3.30)	0.004
Operation, N (%)	817	(4.42)	473	(6.00)	344	(3.25)	0.131	373	(4.81)	187	(4.82)	186	(4.80)	0.001
Anti-cancer drugs, N (%)*	9099	(49.28)	6560	(83.22)	2539	(23.99)	1.476	4864	(62.75)	2579	(66.54)	2285	(58.95)	0.157
CV medications														
Anti-arrhythmia drugs, N (%)**	398	(2.16)	145	(1.84)	253	(2.39)	0.038	162	(2.09)	80	(2.06)	82	(2.12)	0.004
ACEIs/ARBs, N (%)	4140	(22.42)	1184	(15.02)	2956	(27.93)	0.318	1495	(19.29)	732	(18.89)	763	(19.69)	0.020
Beta blockers, N (%)	3803	(20.60)	1462	(18.55)	2341	(22.12)	0.089	1463	(18.87)	724	(18.68)	739	(19.07)	0.010
Anti-platelet agents, N (%)	2785	(15.08)	898	(11.39)	1887	(17.83)	0.183	1055	(13.61)	528	(13.62)	527	(13.60)	0.001
Anti-coagulants, N (%)	364	(1.97)	128	(1.62)	236	(2.23)	0.044	153	(1.97)	73	(1.88)	80	(2.06)	0.013
Statins, N (%)	1494	(8.09)	235	(2.98)	1259	(11.90)	0.345	380	(4.90)	187	(4.82)	193	(4.98)	0.007
Digoxin, N (%)	587	(3.18)	363	(4.60)	224	(2.12)	0.138	244	(3.15)	129	(3.33)	115	(2.97)	0.021
MRAs, N (%)	624	(3.38)	212	(2.69)	412	(3.89)	0.068	248	(3.20)	115	(2.97)	133	(3.43)	0.026
Comorbidities														
CAD, N (%)	2553	(13.83)	1042	(13.22)	1511	(14.28)	0.031	1061	(13.69)	512	(13.21)	549	(14.16)	0.028
PAD, N (%)	357	(1.93)	119	(1.51)	238	(2.25)	0.055	143	(1.84)	69	(1.78)	74	(1.91)	0.010
Hypertension, N (%)	7927	(42.93)	2656	(33.69)	5271	(49.81)	0.331	3202	(41.31)	1566	(40.40)	1636	(42.21)	0.037
Diabetes mellitus, N (%)	3361	(18.20)	1155	(14.65)	2206	(20.85)	0.163	1270	(16.38)	638	(16.46)	632	(16.31)	0.004
Hyperlipidemia, N (%)	2764	(14.97)	675	(8.56)	2089	(19.74)	0.325	897	(11.57)	451	(11.64)	446	(11.51)	0.004
Valve disease, N (%)	523	(2.83)	175	(2.22)	348	(3.29)	0.065	209	(2.70)	104	(2.68)	105	(2.71)	0.002
COPD, N (%)	4917	(26.63)	2778	(35.24)	2139	(20.21)	0.341	2279	(29.40)	1151	(29.70)	1128	(29.10)	0.013
Asthma, N (%)	1861	(10.08)	932	(11.82)	929	(8.78)	0.100	789	(10.18)	408	(10.53)	381	(9.83)	0.023
CKD, N (%)	1019	(5.52)	299	(3.79)	720	(6.80)	0.135	371	(4.79)	179	(4.62)	192	(4.95)	0.016
ESRD, N (%)	26	(0.14)	3	(0.04)	23	(0.22)	0.050	7	(0.09)	3	(0.08)	4	(0.10)	0.009

ASMD, absolute standardized mean difference; CV, cardiovascular; ACEI/ARB, angiotensin-converting enzyme inhibitor/Angiotensin Receptor Blocker; MRA, mineralocorticoid-receptor antagonists; CAD, coronary artery disease; PAD, peripheral artery disease; COPD, chronic obstructive pulmonary disease; CKD, chronic kidney disease; ESRD, end-stage renal disease.

*Anti-cancer drugs is listed in **Supplemental Table 2**.

**Anti-arrhythmia drugs is listed in **Supplemental Table 3**.

### Risks of arrhythmia between patients with NSCLC receiving platinum analogues and those receiving TKIs

**Figure 1:**
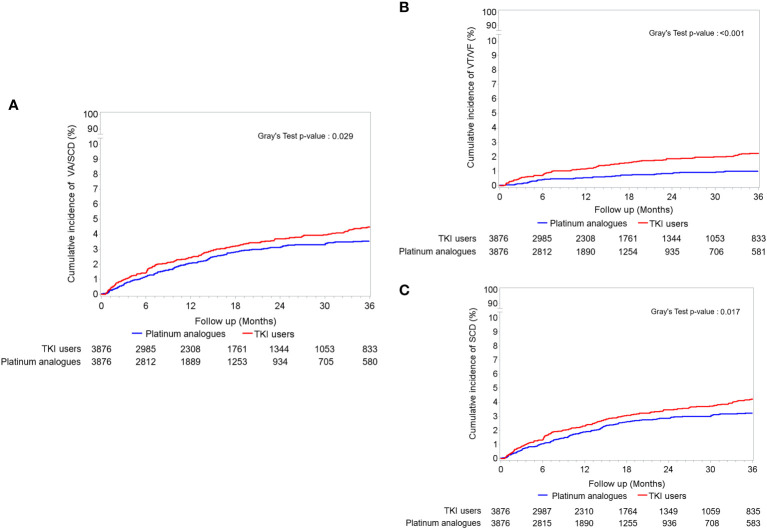
The accumulating incidences of **(A)** the composition endpoint of ventricular arrhythmia (VA) and sudden cardiac death (SCD), **(B)** only VA including ventricular tachycardia (VT)/ventricular fibrillation (VF), and **(C)** only SCD between tyrosine kinase inhibitor (TKI) and platinum analogue users in patients with non-small cell lung cancers (NSCLCs).

During the three-year follow-up period, up to 81.97% of the study population reached the endpoint of mortality (**Table 2**). Compared to patients receiving platinum analogues, those receiving TKIs had a significantly lower risk of death (crude HR: 0.771; CI: 0.733–0.810, p < 0.001). The impact of TKI use on all-cause death remained after adjusting for age, sex, comorbidities, anticancer therapy, and cardiovascular medications (adjusted HR: 0.767; CI: 0.729–0.807, p < 0.001). Cancer was the main cause of death (**Supplemental Table 4**). As shown in **Supplement Table 5**, the time to death interval was prolonged in TKI users compared with that in Platinum analogue users (403.11 ± 291.61 days vs. 342.42 ± 255.26 days). We examined the risk of subsequent arrhythmia, including VA, SCD, and AF. The risk of VA in patients receiving TKIs was significantly higher than that in patients treated with platinum analogues (adjusted HR: 1.987; CI: 1.345–2.935; p < 0.001). Considering that a proportion of patients may die before reaching the arrhythmia endpoint, we adjusted for mortality as a competing risk. Interestingly, we observed significantly increased risks of both VA (adjusted subdistribution HR: 2.328; CI: 1.592–3.404, p < 0.001), SCD (adjusted subdistribution HR: 1.316; CI: 1.041–1.663, p = 0.022), and the composition endpoint of VA/SCD (adjusted subdistribution HR: 1.273; CI: 1.106–1.593, p = 0.036) in TKI users compared with those receiving platinum analogues. In contrast, the risk of AF did not significantly change between patients receiving TKI and those treated with platinum analogues. The time to event intervals of VA, SCD and AF were generally similar between the two groups (**Supplement Table 5**). As depicted in **Figure 1**, the cumulative incidence of VA, SCD, and the composition endpoints of VA/SCD among TKI users was noticeably higher than that among platinum analogue users.

**Table 2:** The crude and adjusted hazard ratio (HR) and subdistribution hazard ratio (sHR) of patients with non-small cell lung cancer (NSCLC) treated with either Platinum analogues or TKIs.

	All, N (%) N=7752		Platinum analogues (Ref.), N (%) N=3876		TKIs, N (%) N=3876		Crude HR (95%CI)	p value	Adjusted HR (95%CI)	p value	Adjusted sHR (95%CI)	p value
Death	6354	(81.97)	3298	(85.09)	3056	(78.84)	0.771 (0.733-0.810)	<0.001	0.767 (0.729-0.807)	<0.001		
VA/SCD	312	(4.02)	137	(3.53)	175	(4.51)	1.068 (0.854-1.337)	0.563	1.079 (0.858-1.357)	0.517	1.273 (1.106-1.593)	0.036
SCD	290	(3.74)	125	(3.22)	165	(4.26)	1.104 (0.874-1.393)	0.407	1.116 (0.879-1.417)	0.367	1.316 (1.041-1.663)	0.022
VA (VT/VF)	124	(1.60)	38	(0.98)	86	(2.22)	1.893 (1.291-2.776)	0.001	1.987 (1.345-2.935)	<0.001	2.328 (1.592-3.404)	<0.001
AF	263	(3.39)	116	(2.99)	147	(3.79)	1.144 (0.896-1.459)	0.281	1.097 (0.854-1.410)	0.468	1.188 (0.920-1.535)	0.186

SCD, sudden cardiac death; VA, ventricular arrhythmia including ventricular tachycardia (VT) and ventricular fibrillation (VF); AF, atrial fibrillation.

Model was adjusted for age, sex, therapies use during (radiotherapy, operation, anti-arrhythmia drugs, anti-cancer drugs), CV medication (ACEI/ARB, beta blocker, anti-platelet agents, anti-coagulants, statins, digoxin, MRA), comorbidities (Coronary artery disease, peripheral artery disease, hypertension, diabetes mellitus, hyperlipidemia, valve disease, chronic obstructive lung disease, asthma, chronic kidney disease, ESRD).

### Subgroup analysis of arrhythmia between patients with NSCLC receiving platinum analogues or TKIs

**Figure 2:**
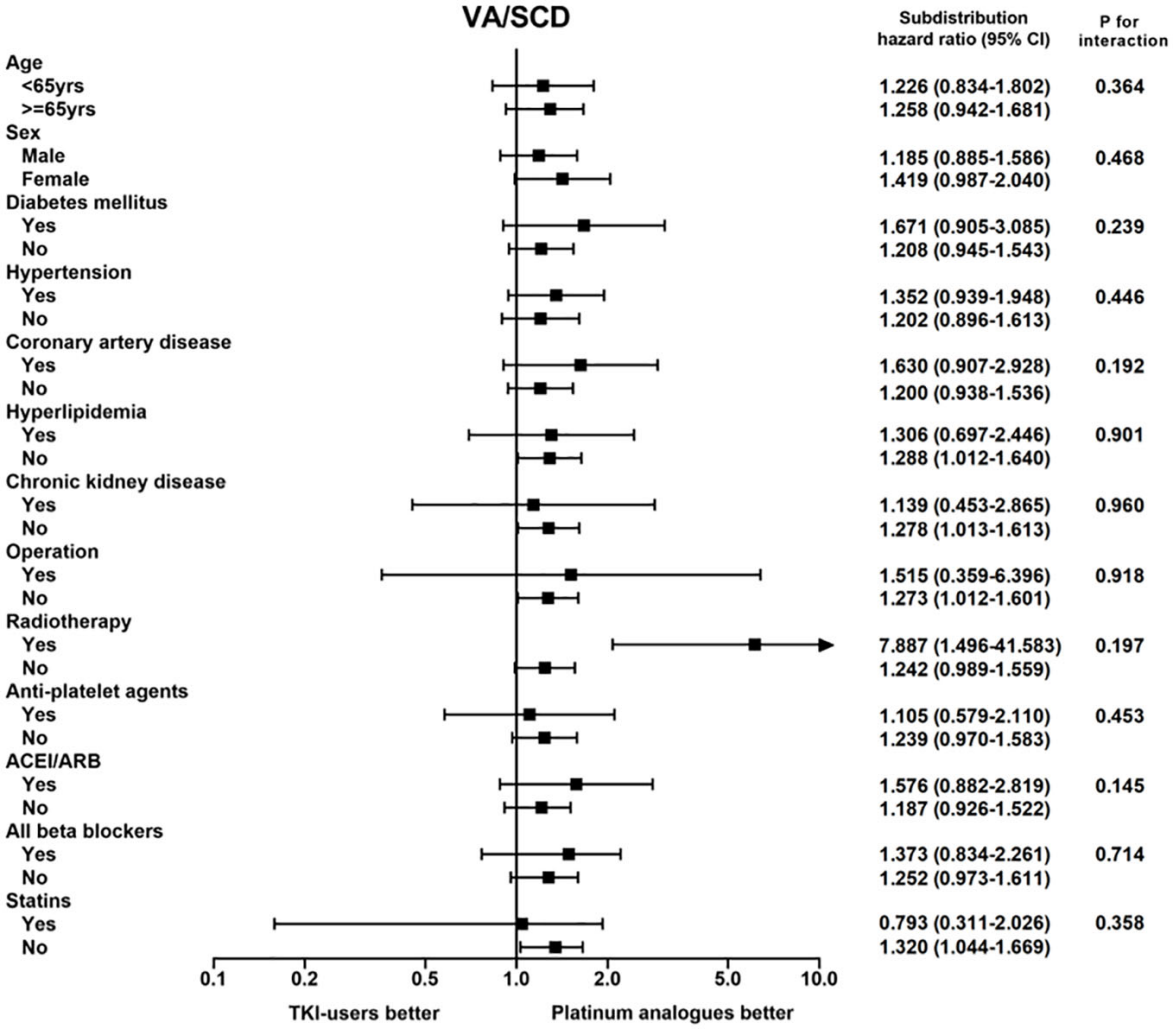
The subgroup analysis of the composition endpoint of ventricular arrhythmia (VA) and sudden cardiac death (SCD) between tyrosine kinase inhibitor (TKI) and platinum analogue users in patients with non-small cell lung cancers (NSCLCs).

As a result of our findings that TKI users had a higher risk of VA and SCD than platinum analogue users, we further investigated whether this phenomenon could be observed in patients with other features. In the subgroup analysis, we discovered that the risk of a composite endpoint of VA and SCD relatively increased in TKI users, regardless of their sex, history of diabetes, hypertension, CAD, or use of cardiovascular medications, compared to platinum analogue users (**Figure 2**). Notably, the increased risks were most significant in patients free from hyperlipidemia, chronic kidney disease, statin use, or undergoing surgery for cancer therapies. In contrast, TKI users who received radiotherapy presented a significantly higher risk of fatal arrhythmia than those treated with platinum analogues. Taken together, we discovered that although patients receiving TKIs had a significantly lower risk of death than those receiving platinum analogues, TKI use was associated with significantly elevated risks of both VA and SCD compared with platinum analogue use. The augmented risk was independent of sex and cardiovascular comorbidities.

## Discussion

Although TKIs have significantly enhanced the survival of patients with NSCLC with EGFR mutations, potential cardiotoxicity, especially fatal arrhythmia, threatens the benefit of long-term mortality and morbidity (9, 10). With a high prevalence of EGFR mutations in Asia, the use of TKIs is higher in Asia than in western countries (8). Hence, it is critical to balance the positive and negative effects of TKIs on survival and cardiovascular outcomes. By analyzing this national cohort, we found that despite a lower probability of mortality in TKI-treated patients with NSCLC than in platinum analogue-treated patients, TKI use was associated with a higher incidence of fatal arrhythmia, including VA and SCD, than platinum analogue use. Notably, the increased risk was independent of cardiovascular risk factors and comorbidities. Given that TKI treatment related QT prolongation and arrhythmias seem to be underestimated owing to inadequate reporting in the post-marketing setting, a strong cooperation between pharmacologists, oncologists and cardiologists and a timely recognition of TKI use related cardiovascular toxicity were crucial (15). Our result sheds light on the fact that during the treatment in patients with NSCLC, clinical practitioners should pay more attention to the potential risks of fatal arrhythmia, while early detection of cardiotoxicities and rapid treatment could increase the advantages of TKI therapy (16, 17).

Cancer therapy-related cardiac dysfunction has detrimental effects on the survival and quality of life of patients with NSCLC (9–11). Before TKIs, platinum-based chemotherapy was the standard treatment for patients with advanced NSCLC, but the systemic use of platinum analogues is often associated with atrial complexes, ventricular premature beats, and bradycardia, which can start immediately during infusion (3, 4). Cases of VA and AF have also been reported. Zellos et al. indicated that among patients receiving hyperthermic cisplatin, the incidence of AF and non-sustained ventricular tachycardia was estimated to be approximately 18.8% and 8%, respectively (18). Platinum analogue-induced electrolyte imbalances, including hypokalemia, hypophosphatemia, hypocalcemia, and hypomagnesemia, have been speculated to be major causes of arrhythmia complications (3, 4). Owing to renal magnesium wasting and decreased intestinal absorption, platinum-induced hypomagnesemia, which has been reported to last for up to six years after cessation of treatment, may cause severe and potentially fatal arrhythmia (4, 19). On the other hand, the development of targeted therapies has significantly improved cancer treatment in recent decades (7, 20). The potential benefit of TKIs on long-term outcomes in patients with EGFR-mutated NSCLC may be constrained by cardiotoxicity associated with off-target effects (9, 10). Given the pivotal role of tyrosine kinases in regulating extracellular signals that control cell growth, differentiation, metabolism, and survival (7, 10, 21), inhibition of tyrosine kinases may result in the development of HF, coronary spasm, and thromboembolic events (22, 23). Mechanistically, EGFR kinase regulates the human ether-a-go-go (hERG) subunit, which conducts the main ventricular repolarization potassium current (IKr) potential during phases 2–3 of the action potential (24, 25). Novo et al, indicated that through the “off-target effect”, TKIs could also work on endothelial cells, which plays a pivotal role in maintaining vascular homeostasis by producing vasoconstrictor and vasodilator substances (26). It result in different type of toxicities, including QT interval prolongation and arrhythmias, compared with traditional chemotherapy (26, 27). Also, using whole-cell patch-clamp technique in guinea pig ventricular myocytes, Jie et al. reported that gefitinib could delay repolarization as well as prolong QTc interval by potently blocking hERG channel (28). Likewise, using a small animal model and primary cardiomyocytes, we previously showed that TKIs suppress the amplitude of the slowly activating delayed-rectifier K + current (IK_S_) in a time- and concentration-dependent manner, which could be an important mechanism underlying changes in QTc intervals (24). To date, there is a lack of an effective method to reverse drug-induced fatal arrhythmia; thus, close monitoring during TKI use is warranted.

In the subgroup analysis, we observed that the risks of VA/SCD relatively increased in TKI users compared to platinum analogue users, independent of their sex, cardiovascular risk factors, or use of cardiovascular drugs. Interestingly, TKI users who received radiotherapy presented a significantly higher risk of fatal arrhythmia than those treated with platinum analogues. As previously reported, radiation therapy may contribute to myocardial fibrosis and autonomic dysfunction, leading to arrhythmia (29, 30). Therefore, combined TKI use and radiotherapy may have a detrimental effect on VA/SCD. However, the risk of AF was not significantly different between patients treated with TKI and those receiving platinum analogues. Tamargo et al. reported a wide range of AF incidence, with cisplatin use ranging from 15% to 32%, whereas the incidence of AF varies among different types of TKIs (31). Ibrutinib, a small-molecule drug that inhibits B-cell proliferation, has been reported to increase the risk of AF, reaching 38% (32). Likewise, the FDA Adverse Events Reporting System has indicated that osimertinib, a third-generation TKI for NSCLC, has fourfold increasing risks of AF compared to the control arm (33). However, regarding the first- and second-generation TKIs included in this study, the AF incidence ranged from 0.43 to 1.79% (34). The structural difference between each drug is plausible to be the reason for the different effects on arrhythmia.

Our study has some limitations. First, owing to the relatively limited survival in advanced NSCLC, patients may die before reaching cardiovascular endpoints. Alternatively, we adjusted for mortality as a competing risk and observed a persistent increase inarrhythmias in TKI users compared with non-users. Second, given that arrhythmias could not only attribute to the sue of TKIs but the effects of NSCLC, such as systemic inflammation, per se, whether the cardiovascular complications that we observed was merely owing to the treatments required more evidences. However, through a careful matching, TKI use was still associated with a higher risk of ventricular arrhythmia than those receiving conventional chemotherapy of platinum analogues in patients with the background of NSCLC. Third, although the control group of platinum users were selected during the time before TKIs were available, there could still be patients subsequently receiving TKIs during the follow-up period after TKIs were launched and those patients who had EGFR mutations. The switch between groups may influence the impact of each drug on the outcomes. Forth, some clinical information such as the QT interval and laboratory data were not available in this cohort. Last, osimertinib has been shown to have an increased risk of cardiotoxicity compared with first-generation TKIs (33, 35). Osimertinib-related information was not included in the study because it was not yet available in the NHIRD. Third, TKI use depends on EGFR mutations, which may be an inherent overall survival difference between TKI and platinum analogue groups. To overcome selection bias, we randomly selected a control group at the time when TKIs were not yet available to match with a study group of TKI use.

## Conclusions

The development of TKI has significantly improved the treatment of patients with NSCLC; however, its benefits may be attenuated by off-target effects related to arrhythmia. In this nationwide cohort, we identified that TKI was associated with a lower mortality risk but a higher risk of fatal arrhythmia than platinum analogues. Here, we provide evidence for ongoing surveillance of cardiovascular problems during TKI use. Further research is required to validate our findings.

## Data availability statement

The datasets presented in this article are not readily available because: The original data belongs to NHIRD. Requests to access the datasets should be directed to cmcvecho3@gmail.com.

## Ethics statement

The studies involving human participants were reviewed and approved by IRB A-EX-111-003; CV code: 10406-E01. Written informed consent for participation was not required for this study in accordance with the national legislation and the institutional requirements.

## Author contributions

Conceptualization: W-TC, S-HL, Y-HL, H-WL. Methodology: W-TC, S-HL and H-WL. Software: S-HL and H-WL. Validation: W-TC, S-HL, Y-HL, H-WL. Formal analysis: S-HL and H-WL. Investigation: W-TC, S-HL, Y-HL, H-WL. Resources: Y-HL. Data curation: S-HL and H-WL. Writing—original draft preparation: W-TC. Writing—review and editing: W-TC, Y-HL, H-WL. Visualization: W-TC, S-HL, Y-HL, T-CC, H-WL. Supervision: S-HL, T-CC, and Y-HL. Project administration: W-TC and Y-HL. Funding acquisition: W-TC and Y-HL. All authors have read and agreed to the published version of the manuscript. All authors contributed to the article and approved the submitted version.

## Supplementary material

Supplementary Figure 1 The flowchart of study design.

## References

[1] Group NM-AC. Chemotherapy in addition to supportive care improves survival in advanced non-small-cell lung cancer: a systematic review and meta-analysis of individual patient data from 16 randomized controlled trials. J Clin Oncol (2008) 26:4617–25. doi: 10.1200/JCO.2008.17.7162 PMC2653127 18678835

[2] Lin JJ Cardarella S Lydon CA Dahlberg SE Jackman DM Janne PA. Five-year survival in EGFR-mutant metastatic lung adenocarcinoma treated with EGFR-TKIs. J Thorac Oncol (2016) 11:556–65. doi: 10.1016/j.jtho.2015.12.103 PMC4979601 26724471

[3] Avan A Postma TJ Ceresa C Avan A Cavaletti G Giovannetti E. Platinum-induced neurotoxicity and preventive strategies: past, present, and future. Oncologist (2015) 20:411–32. doi: 10.1634/theoncologist.2014-0044 PMC4391771 25765877

[4] Ferroni P Della-Morte D Palmirotta R McClendon M Testa G Abete P. Platinum-based compounds and risk for cardiovascular toxicity in the elderly: role of the antioxidants in chemoprevention. Rejuvenation Res (2011) 14:293–308. doi: 10.1089/rej.2010.1141 21595514

[5] Lassen U Osterlind K Hansen M Dombernowsky P Bergman B Hansen HH. Long-term survival in small-cell lung cancer: posttreatment characteristics in patients surviving 5 to 18+ years–an analysis of 1,714 consecutive patients. J Clin Oncol (1995) 13:1215–20. doi: 10.1200/JCO.1995.13.5.1215 7738624

[6] Heuckmann JM Rauh D Thomas RK. Epidermal growth factor receptor (EGFR) signaling and covalent EGFR inhibition in lung cancer. J Clin Oncol (2012) 30:3417–20. doi: 10.1200/JCO.2012.43.1825 22915655

[7] Batson S Mitchell SA Windisch R Damonte E Munk VC Reguart N. Tyrosine kinase inhibitor combination therapy in first-line treatment of non-small-cell lung cancer: systematic review and network meta-analysis. Onco Targets Ther (2017) 10:2473–82. doi: 10.2147/OTT.S134382 PMC5426468 28503070

[8] Shi Y Au JS Thongprasert S Srinivasan S Tsai CM Khoa MT. A prospective, molecular epidemiology study of EGFR mutations in Asian patients with advanced non-small-cell lung cancer of adenocarcinoma histology (PIONEER). J Thorac Oncol (2014) 9:154–62. doi: 10.1097/JTO.0000000000000033 PMC4132036 24419411

[9] Chen MH Kerkela R Force T. Mechanisms of cardiac dysfunction associated with tyrosine kinase inhibitor cancer therapeutics. Circulation (2008) 118:84–95. doi: 10.1161/CIRCULATIONAHA.108.776831 18591451 PMC2735334

[10] Orphanos GS Ioannidis GN Ardavanis AG. Cardiotoxicity induced by tyrosine kinase inhibitors. Acta Oncol (2009) 48:964–70. doi: 10.1080/02841860903229124 19734999

[11] Waliany S Zhu H Wakelee H Padda SK Das M Ramchandran K. Pharmacovigilance analysis of cardiac toxicities associated with targeted therapies for metastatic NSCLC. J Thorac Oncol (2021) 16:2029–39. doi: 10.1016/j.jtho.2021.07.030 34418561

[12] Cheng CL Kao YH Lin SJ Lee CH Lai ML. Validation of the national health insurance research database with ischemic stroke cases in Taiwan. Pharmacoepidemiol Drug Saf. (2011) 20:236–42. doi: 10.1002/pds.2087 21351304

[13] Huang K Lin FJ Ou HT Hsu CN Huang LY Wang CC. Building an active medical product safety surveillance system in Taiwan: adaptation of the U.S. sentinel system common data model structure to the national health insurance research database in Taiwan. Pharmacoepidemiol Drug Saf. (2021) 30:97–101. doi: 10.1002/pds.5168 33146908

[14] Austin PC. An introduction to propensity score methods for reducing the effects of confounding in observational studies. Multivariate Behav Res (2011) 46:399–424. doi: 10.1080/00273171.2011.568786 21818162 PMC3144483

[15] Bonura F Di Lisi D Novo S D'Alessandro N. Timely recognition of cardiovascular toxicity by anticancer agents: a common objective of the pharmacologist, oncologist and cardiologist. Cardiovasc Toxicol (2012) 12:93–107. doi: 10.1007/s12012-011-9141-z 21894547

[16] Albini A Pennesi G Donatelli F Cammarota R De Flora S Noonan DM. Cardiotoxicity of anticancer drugs: the need for cardio-oncology and cardio-oncological prevention. J Natl Cancer Inst (2010) 102:14–25. doi: 10.1093/jnci/djp440 20007921 PMC2802286

[17] Khouri MG Klein MR Velazquez EJ Jones LW. Current and emerging modalities for detection of cardiotoxicity in cardio-oncology. Future Cardiol (2015) 11:471–84. doi: 10.2217/fca.15.16 PMC5558528 26235924

[18] Zellos L Richards WG Capalbo L Jaklitsch MT Chirieac LR Johnson BE. A phase I study of extrapleural pneumonectomy and intracavitary intraoperative hyperthermic cisplatin with amifostine cytoprotection for malignant pleural mesothelioma. J Thorac Cardiovasc Surg (2009) 137:453–8. doi: 10.1016/j.jtcvs.2008.07.055 19185169

[19] Oronsky B Caroen S Oronsky A Dobalian VE Oronsky N Lybeck M. Electrolyte disorders with platinum-based chemotherapy: mechanisms, manifestations and management. Cancer Chemother Pharmacol (2017) 80:895–907. doi: 10.1007/s00280-017-3392-8 28730291 PMC5676816

[20] Alanazi A Yunusa I Elenizi K Alzarea AI. Efficacy and safety of tyrosine kinase inhibitors in advanced non-small-cell lung cancer harboring epidermal growth factor receptor mutation: a network meta-analysis. Lung Cancer Manage (2020) 10:LMT43. doi: 10.2217/lmt-2020-0011 PMC7724735 33318755

[21] Du Z Lovly CM. Mechanisms of receptor tyrosine kinase activation in cancer. Mol Cancer. (2018) 17:58. doi: 10.1186/s12943-018-0782-4 29455648 PMC5817791

[22] Grisanti LA Talarico JA Carter RL Yu JE Repas AA Radcliffe SW. Beta-adrenergic receptor-mediated transactivation of epidermal growth factor receptor decreases cardiomyocyte apoptosis through differential subcellular activation of ERK1/2 and akt. J Mol Cell Cardiol (2014) 72:39–51. doi: 10.1016/j.yjmcc.2014.02.009 24566221 PMC4037368

[23] Noma T Lemaire A Naga Prasad SV Barki-Harrington L Tilley DG Chen J. Beta-arrestin-mediated beta1-adrenergic receptor transactivation of the EGFR confers cardioprotection. J Clin Invest. (2007) 117:2445–58. doi: 10.1172/JCI31901 PMC1952636 17786238

[24] Chang WT Liu PY Lee K Feng YH Wu SN. Differential inhibitory actions of multitargeted tyrosine kinase inhibitors on different ionic current types in cardiomyocytes. Int J Mol Sci (2020) 21:1672. doi: 10.3390/ijms21051672 32121388 PMC7084345

[25] Missan S Linsdell P McDonald TF. Tyrosine kinase and phosphatase regulation of slow delayed-rectifier k+ current in guinea-pig ventricular myocytes. J Physiol (2006) 573:469–82. doi: 10.1113/jphysiol.2005.104422 PMC1779722 16581870

[26] Novo G Di Lisi D Bronte E Macaione F Accurso V Badalamenti G. Cardiovascular toxicity in cancer patients treated with tyrosine kinase inhibitors: a real-world single-center experience. Oncology (2020) 98:445–51. doi: 10.1159/000505486 32348984

[27] Di Lisi D Madonna R Zito C Bronte E Badalamenti G Parrella P. Anticancer therapy-induced vascular toxicity: VEGF inhibition and beyond. Int J Cardiol (2017) 227:11–7. doi: 10.1016/j.ijcard.2016.11.174 27866063

[28] Jie LJ Li YD Zhang HQ Mao L Xie HB Zhou FG. Mechanisms of gefitinib-induced QT prolongation. Eur J Pharmacol (2021) 910:174441. doi: 10.1016/j.ejphar.2021.174441 34474028

[29] Belzile-Dugas E Eisenberg MJ. Radiation-induced cardiovascular disease: review of an underrecognized pathology. J Am Heart Assoc (2021) 10:e021686. doi: 10.1161/JAHA.121.021686 34482706 PMC8649542

[30] Chang HM Okwuosa TM Scarabelli T Moudgil R Yeh ETH. Cardiovascular complications of cancer therapy: best practices in diagnosis, prevention, and management: part 2. J Am Coll Cardiol (2017) 70:2552–65. doi: 10.1016/j.jacc.2017.09.1095 PMC5825188 29145955

[31] Tamargo J Caballero R Delpon E. Drug-induced atrial fibrillation. Expert Opin Drug Saf. (2012) 11:615–34. doi: 10.1517/14740338.2012.698609 22724662

[32] Kornej J Borschel CS Benjamin EJ Schnabel RB. Epidemiology of atrial fibrillation in the 21st century: novel methods and new insights. Circ Res (2020) 127:4–20. doi: 10.1161/CIRCRESAHA.120.316340 32716709 PMC7577553

[33] Anand K Ensor J Trachtenberg B Bernicker EH. Osimertinib-induced cardiotoxicity: a retrospective review of the FDA adverse events reporting system (FAERS). JACC CardioOncol. (2019) 1:172–8. doi: 10.1016/j.jaccao.2019.10.006 PMC8352117 34396179

[34] Cheng M Yang F Liu J Yang D Zhang S Yu Y. Tyrosine kinase inhibitors-induced arrhythmias: from molecular mechanisms, pharmacokinetics to therapeutic strategies. Front Cardiovasc Med (2021) 8:758010. doi: 10.3389/fcvm.2021.758010 34869670 PMC8639698

[35] Ewer MS Tekumalla SH Walding A Atuah KN. Cardiac safety of osimertinib: a review of data. J Clin Oncol (2021) 39:328–37. doi: 10.1200/JCO.20.01171 PMC8078322 33356419

