# Supplementary material for: The association between tyrosine kinase inhibitors and fatal arrhythmia in patients with non-small cell lung cancer in Taiwan
Source: Front Oncol. 2023 Apr 17;13:1172036. doi: 10.3389/fonc.2023.1172036 (PMC10150998; doi:10.3389/fonc.2023.1172036)
Supplement: Supplementary file 1 [file Table_1.docx]

| Supplemental Table 1. ICD-9 and ICD-10 codes   \| Disease \| ICD-9 Codes \| ICD-10 Codes \| \| --- \| --- \| --- \| \| Lung cancer \| 162 \| C34 \| \| Outcome \| \| \| \| Atrial fibrillation (AF) \| 427.31,427.32 \| I48.xx \| \| Sudden Cardiac Death (SCD) \| Instantaneous death (798.1; 798.9, V12.53) 798.2, 427.5 \| I46.2, I46.8, I46.9, R99 \| \| Ventricular arrhythmia (VA) including ventricular tachycardia and ventricular fibrillation \| paroxysmal ventricular tachycardia (427.1); ventricular fibrillation and flutter (427.4);  cardiac arrest (427.5) \| I47.0, I47.2, I49.3, I49.01, I49.02 \| \| Comorbidities \| \| \| \| Coronary artery disease \| 410, 411, 412, 413, 414 \| I20, I21, I22, I24, I25 \| \| Peripheral artery disease \| 440, 443, 444, 447.8, 447.9 \| I70.2-I70.9, I71, I73.9, I74.2, I74.3, I74.4, I74.5, I77.89, I77.9 \| \| Hypertension \| 401, 402, 403, 404, 405 \| I10, I11.0, I11.9, I12.0, I12.9, I13.0, I13.2, I13.11, I15, N26.2 \| \| Diabetes mellitus \| 250 \| E08, E09, E11, E13 \| \| Hyperlipidemia \| 272 \| E78 \| \| Valve disorders \| 394-397, 424.0, 424.1, 424.2, 424.3 \| I05, I06, I07, I08, I09, I34-I37 \| \| Chronic obstructive pulmonary disease \| 491, 492, 494, 495, 496 \| J41, J42, J43, J44, J47, J67 \| \| Asthma \| 493 \| J45 \| \| Atrial fibrillation \| 427.31, 427.32 \| I48 \| \| Chronic kidney disease \| 580-589, 403, 404, 585, V45.1, V56 \| I12, I13, N02, N03, N04, N05, N06, N07, N08, N11, N14, N17, N18, N19, N29, O10.2, O10.3, Q61, Z49, Z99.2 \| \| ESRD \| 585 \| N18.6; Z99.2 \| \| Procedure Code \| \| \| \| Radiotherapy \| V58.0 \| Z51.0 \| \| Operations \| 67023B (single lobectomy), 67042B(Bilobectomy), 67050B (Thoracoscopic Lobectomy) \| \|   Abbreviations as listed in Table 1 |
| --- | --- | --- | --- | --- | --- | --- | --- | --- | --- | --- | --- | --- | --- | --- | --- | --- | --- | --- | --- | --- | --- | --- | --- | --- | --- | --- | --- | --- | --- | --- | --- | --- | --- | --- | --- | --- | --- | --- | --- | --- | --- | --- | --- | --- | --- | --- | --- | --- | --- | --- | --- | --- | --- | --- | --- | --- | --- | --- | --- | --- | --- | --- | --- |

| Supplemental Table 2. The lists of Anti-cancer drug uses of patients with non-small cell lung cancer (NSCLC) treated with either Platinum analogues or TKIs before and after propensity score matching | | | | | | | | | | | | | | | |
| --- | --- | --- | --- | --- | --- | --- | --- | --- | --- | --- | --- | --- | --- | --- | --- |
|  | Before propensity score matching | | | | | | |  | After propensity score matching | | | | | | |
|  | Total  N=18465 | | Platinum analogues  N=7883 | | TKIs  N=10582 | | ASMD |  | Total  N=7752 | | Platinum analogues  N=3876 | | TKIs  N=3876 | | ASMD |
| Anti-cancer drugs |  |  |  |  |  |  |  |  |  |  |  |  |  |  |  |
| Gemcitabine, N(%) | 4525 | (24.51) | 4037 | (51.21) | 488 | (4.61) | 1.216 |  | 2088 | (26.93) | 1644 | (42.41) | 444 | (11.46) | 0.745 |
| Taxanes, N(%) | 1658 | (8.98) | 1249 | (15.84) | 409 | (3.87) | 0.410 |  | 835 | (10.77) | 448 | (11.56) | 387 | (9.98) | 0.051 |
| Docetaxel, N(%) | 711 | (3.85) | 402 | (5.10) | 309 | (2.92) | 0.111 |  | 433 | (5.59) | 138 | (3.56) | 295 | (7.61) | 0.177 |
| Paclitaxel, N(%) | 954 | (5.17) | 849 | (10.77) | 105 | (0.99) | 0.425 |  | 408 | (5.26) | 311 | (8.02) | 97 | (2.50) | 0.249 |
| Vinorelbine, N(%) | 3121 | (16.90) | 1607 | (20.39) | 1514 | (14.31) | 0.161 |  | 1952 | (25.18) | 615 | (15.87) | 1337 | (34.49) | 0.439 |
| Pemetrexed, N(%) | 312 | (1.69) | 0 | (0) | 312 | (2.95) | 0.247 |  | 287 | (3.70) | 0 | (0) | 287 | (7.40) | 0.400 |

Abbreviations as listed in Table 1

| Supplemental Table 3. The lists of anti-arrhythmia drug uses of patients with non-small cell lung cancer (NSCLC) treated with either Platinum analogues or TKIs before and after propensity score matching. | | | | | | | | | | | | | | | |
| --- | --- | --- | --- | --- | --- | --- | --- | --- | --- | --- | --- | --- | --- | --- | --- |
|  | Before propensity score matching | | | | | | |  | After propensity score matching | | | | | | |
|  | Total  N=18465 | | Platinum analogues  N=7883 | | TKIs  N=10582 | | ASMD |  | Total  N=7752 | | Platinum analogues  N=3876 | | TKIs  N=3876 | | ASMD |
| Anti-arrhythmia drugs, N(%) | | | | | | | | | | | | | | | |
| Amiodarone, N(%) | 310 | (1.68) | 107 | (1.36) | 203 | (1.92) | 0.044 |  | 125 | (1.61) | 55 | (1.42) | 70 | (1.81) | 0.031 |
| Dronedarone, N(%) | 8 | (0.04) | 0 | (0) | 8 | (0.08) | 0.039 |  | 0 | (0) | 0 | (0) | 0 | (0) | 0.000 |
| Propafenone, N(%) | 73 | (0.40) | 27 | (0.34) | 46 | (0.43) | 0.015 |  | 34 | (0.44) | 20 | (0.52) | 14 | (0.36) | 0.023 |
| Flecainide, N(%) | 0 | (0) | 0 | (0) | 0 | (0) | 0.000 |  | 0 | (0) | 0 | (0) | 0 | (0) | 0.000 |
| Quinidine, N(%) | 20 (0.11) | | | | | | 0.042 |  | 12 (0.15) | | | | | | 0.066 |
| Procainamide, N(%) | 3 | (0.02) | 3 | (0.04) | 0 | (0) | 0.028 |  | 0 | (0) | 0 | (0) | 0 | (0) | 0.000 |

Abbreviations as listed in Table 1

| Supplemental Table 4. The reason of death among NSCLC patients receiving either TKIs or Platinum analogues | | | |
| --- | --- | --- | --- |
|  | Total  N=7752 | Platinum analogues  N=3876 | TKIs  N=3876 |
| Cancer death | 6147 (79.30) | 3218 (83.02) | 2929 (75.57) |
| Cardiovascular death | 46 (0.59) | 14 (0.36) | 32 (0.83) |
| Others | 161 (2.08) | 66 (1.70) | 95 (2.45) |

Abbreviations as listed in Table 1

| Supplemental Table 5. Time to event intervals | | |
| --- | --- | --- |
| Days (mean±SD) | Platinum analogues | TKIs |
| Death | 342.42 ± 255.26 | 403.11 ± 291.61 |
| VA/SCD | 344.89 ± 257.64 | 389.06 ± 317.51 |
| SCD | 344.48 ± 258.13 | 391.59 ± 318.93 |
| VA (VT/VF) | 362.74 ± 279.79 | 381.99 ± 311.13 |
| AF | 198.18 ± 207.51 | 246.40 ± 277.90 |

Abbreviations as listed in Table 1
