# Supplementary figures and images for: The association between tyrosine kinase inhibitors and fatal arrhythmia in patients with non-small cell lung cancer in Taiwan
Source: Front Oncol. 2023 Apr 17;13:1172036. doi: 10.3389/fonc.2023.1172036 (PMC10150998; doi:10.3389/fonc.2023.1172036)

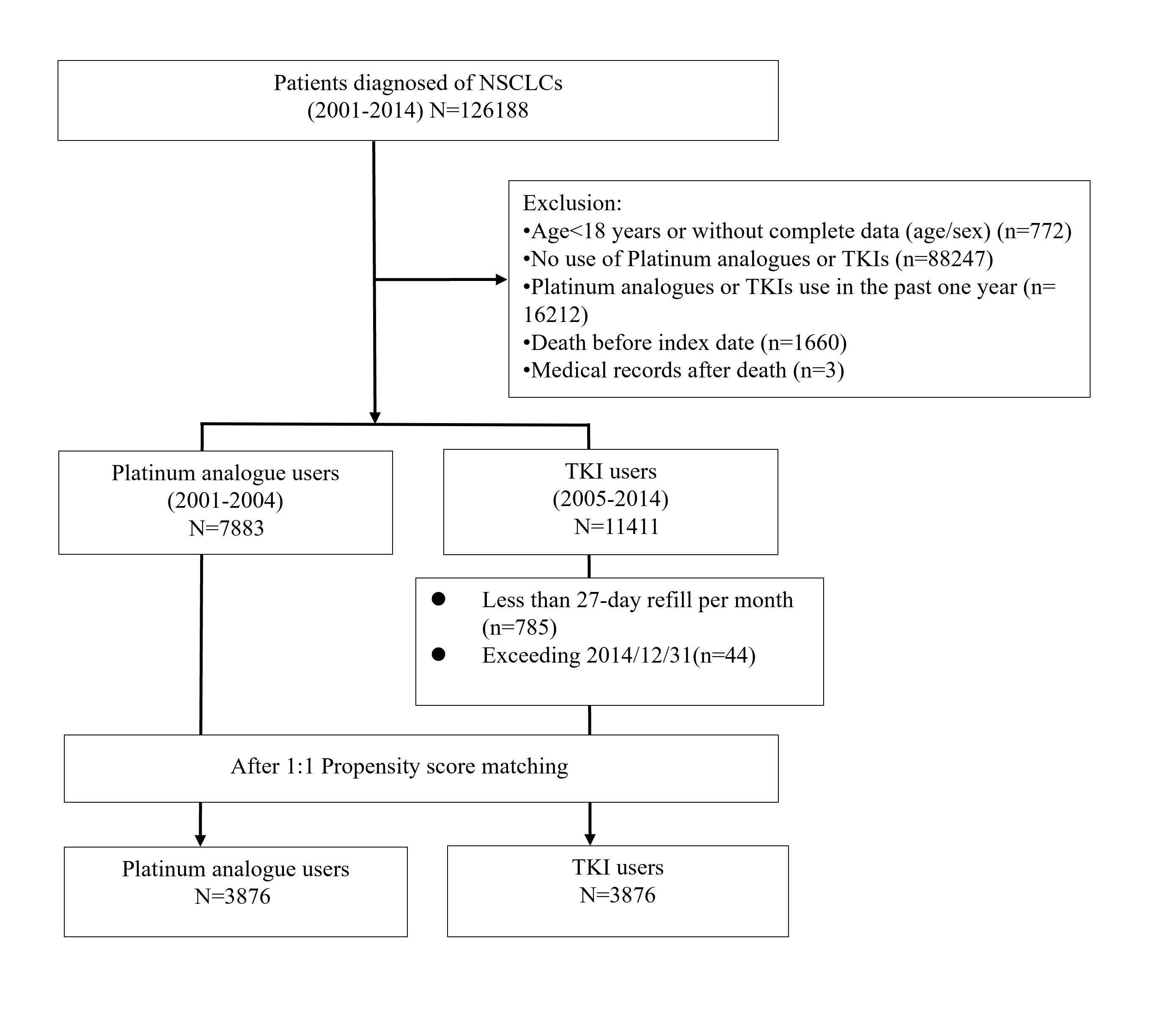

Supplement: Supplementary Figure 1 — The flowchart of study design. [file Image_1.tif]
